# The genome sequence of the common malachite beetle, *Malachius bipustulatus *(Linnaeus, 1758)

**DOI:** 10.12688/wellcomeopenres.17381.1

**Published:** 2021-11-25

**Authors:** Liam M. Crowley, University of Oxford and Wytham Woods Genome Acquisition Lab, Darwin Tree of Life Barcoding collective, Wellcome Sanger Institute Tree of Life programme, Wellcome Sanger Institute Scientific Operations: DNA Pipelines collective, Tree of Life Core Informatics collective, Darwin Tree of Life Consortium

**Affiliations:** 1 Department of Zoology, University of Oxford, Oxford, UK

**Keywords:** Malachius bipustulatus, common malachite beetle, genome sequence, chromosomal, Coleoptera

## Abstract

We present a genome assembly from an individual female *Malachius bipustulatus *(the common malachite beetle; Arthropoda; Insecta; Coleoptera; Melyridae). The genome sequence is 544 megabases in span. The majority (99.70%) of the assembly is scaffolded into 10 chromosomal pseudomolecules, with the X sex chromosome assembled.

## Species taxonomy

Eukaryota; Metazoa; Ecdysozoa; Arthropoda; Hexapoda; Insecta; Pterygota; Neoptera; Endopterygota; Coleoptera; Polyphaga; Cucujiformia; Melyridae; Malachiinae; Malachius; *Malachius bipustulatus* (Linnaeus, 1758) (NCBI:txid41139).

## Background

The common malachite beetle, *Malachius bipustulatus*, is a common soft-winged flower beetle. It is widespread and common throughout Europe and western Asia. In the UK, it is common in lowland grassland and agricultural land across England and Wales, becoming scarcer further north into Scotland. It is metallic green with red tips to the elytra and anterior pronotal angles. It is larger (5.5–6 mm) than the similar *Cordylepherus viridis,* which has a less vivid green colouration and lacks reddened anterior pronotal angles. Adults possess bright red eversible sacs called cocardes along the thorax and abdomen that emit defensive odours, which are readily produced when the beetle is alarmed. It is univoltine, with adults emerging from April/May, and may be commonly encountered on flowers throughout the summer where they feed on pollen and nectar as well as small invertebrates. The strong association of *M. bipustulatus* with flowers may lead to the species acting as an incidental minor pollinator ( Gutowski, 1990). As generalist predators, they may also confer some degree of biological pest control ( Malschi & Others, 2000). Males produce pheromone secretions from glands on the head in between the antennal insertions. These pheromones are transferred to lobes on the inner margin of antennomeres 2–4, with the antennae held forward to attract females. Females feed on these secretions during courtship. Eggs are laid in bark crevices and low vegetation. The larvae are predatory and are relatively long-legged, active hunters that develop throughout the summer ( Gradwell, 1957). Adults may persist until late summer.

## Genome sequence report

**Figure 1.**
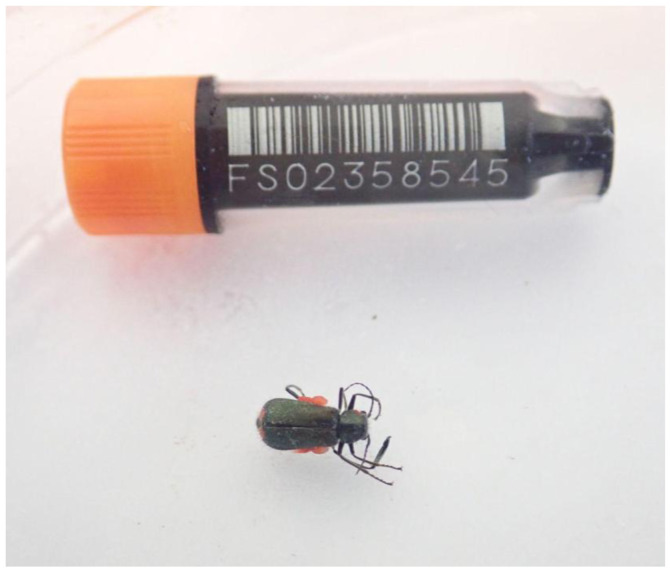
Image of the *Malachius bipustulatus* specimen (icMalBipu1) used for genome sequencing. Image taken during preservation and processing, with 43.9 mm FluidX sample tube shown above.

The genome was sequenced from one female *M. bipustulatus* ( Figure 1) collected from Wytham Woods, Oxfordshire (biological vice-county: Berkshire), UK (latitude 51.768, longitude -0.339). A total of 32-fold coverage in Pacific Biosciences single-molecule long reads and 65-fold coverage in 10X Genomics read clouds were generated. Primary assembly contigs were scaffolded with chromosome conformation Hi-C data. Manual assembly curation corrected 244 missing/misjoins and removed 69 haplotypic duplications, reducing the assembly length by 1.62% and the scaffold number by 66.23%, and increasing the scaffold N50 by 45.84%.

**Figure 2.**
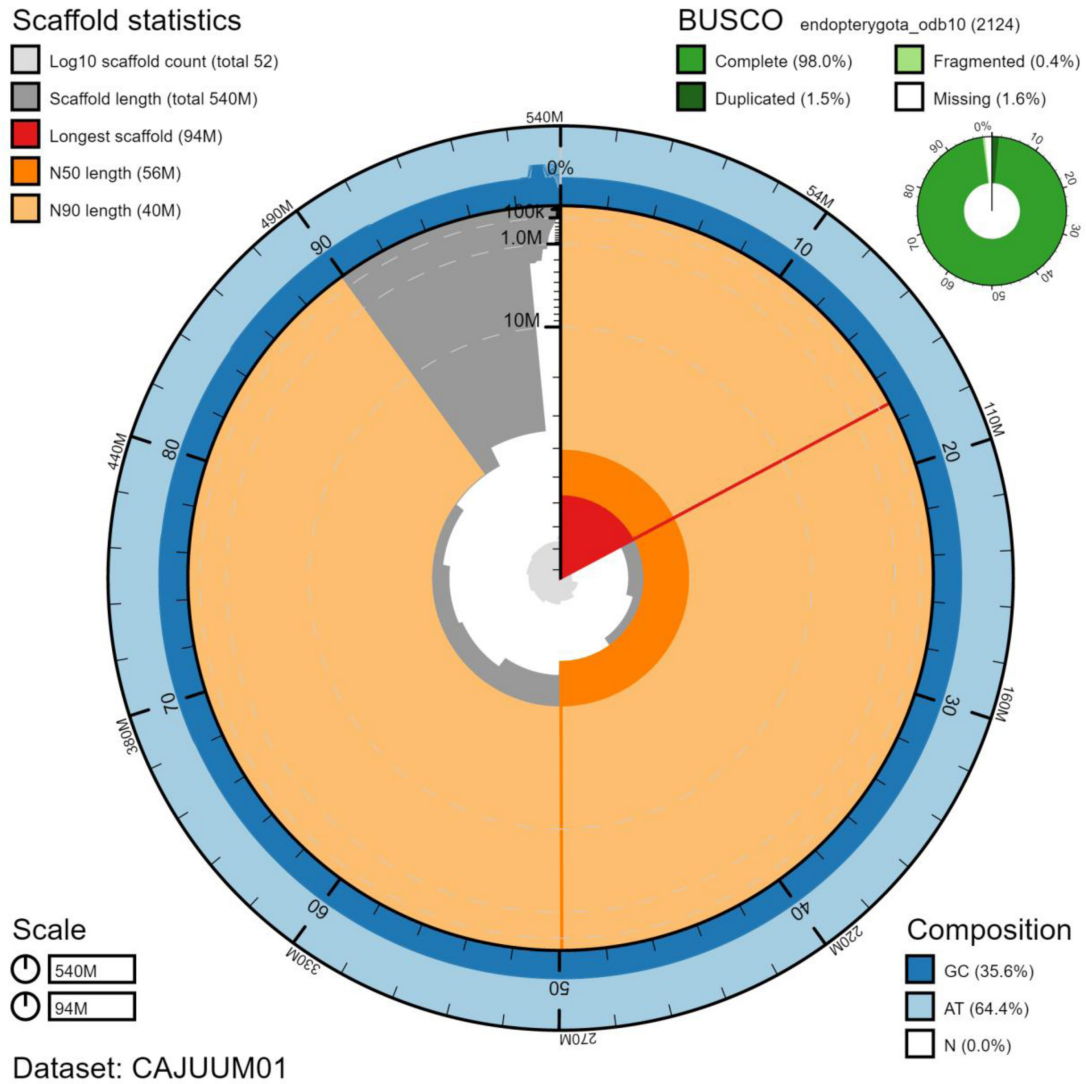
Genome assembly of *Malachius bipustulatus*, icMalBipu1.1: metrics. The BlobToolKit Snailplot shows N50 metrics and BUSCO gene completeness. The main plot is divided into 1,000 size-ordered bins around the circumference with each bin representing 0.1% of the 544,256,285 bp assembly. The distribution of chromosome lengths is shown in dark grey with the plot radius scaled to the longest chromosome present in the assembly (94,150,490 bp, shown in red). Orange and pale-orange arcs show the N50 and N90 chromosome lengths (56,302,816 and 40,414,116 bp), respectively. The pale grey spiral shows the cumulative chromosome count on a log scale with white scale lines showing successive orders of magnitude. The blue and pale-blue area around the outside of the plot shows the distribution of GC, AT and N percentages in the same bins as the inner plot. A summary of complete, fragmented, duplicated and missing BUSCO genes in the endopterygota_odb10 set is shown in the top right. An interactive version of this figure is available at https://blobtoolkit.genomehubs.org/view/icMalBipu1.1/dataset/CAJUUM01/snail.

The final assembly has a total length of 544 Mb in 51 sequence scaffolds with a scaffold N50 of 56.3 Mb ( Table 1). The majority, 99.70%, of the assembly sequence was assigned to 10 chromosomal-level scaffolds, representing 9 autosomes (numbered by sequence length), and the X sex chromosome ( Figure 2– Figure 5; Table 2). Due to ambiguous Hi-C signal and lack of additional evidence to support accurate placement, several scaffolds that associate with chromosome X as evidenced by Hi-C data have been submitted as unlocalised. These components are labelled SUPER_X_unloc_1-4. The assembly has a BUSCO v5.1.2 ( Manni *et al.,* 2021) completeness of 98.0% (single 96.5%, duplicated 1.5%) using the endopterygota_odb10 reference set. While not fully phased, the assembly deposited is of one haplotype. Contigs corresponding to the second haplotype have also been deposited.

**Table 1.** Genome data for *Malachius bipustulatus*, icMalBipu1.1.

*Project accession data*	
Assembly identifier	icMalBipu1.1
Species	*Malachius bipustulatus*
Specimen	icMalBipu1
NCBI taxonomy ID	NCBI:txid295700
BioProject	PRJEB45121
BioSample ID	SAMEA7520537
Isolate information	Female, whole organism
*Raw data accessions*	
PacificBiosciences SEQUEL II	ERR6436375
10X Genomics Illumina	ERR6054777-ERR6054780
Hi-C Illumina	ERR6054776
*Genome assembly*	
Assembly accession	GCA_910589415.1
Accession of alternate haplotype	GCA_910589365.1
Span (Mb)	544
Number of contigs	244
Contig N50 length (Mb)	5.7
Number of scaffolds	51
Scaffold N50 length (Mb)	56.3
Longest scaffold (Mb)	94.2
BUSCO * genome score	C:98.0%[S:96.5%,D:1.5%], F:0.4%,M:1.6%,n:2124

*BUSCO scores based on the endopterygota_odb10 BUSCO set using v5.1.2. C= complete [S= single copy, D=duplicated], F=fragmented, M=missing, n=number of orthologues in comparison. A full set of BUSCO scores is available at https://blobtoolkit.genomehubs.org/view/icMalBipu1.1/dataset/CAJUUM01/busco.

**Figure 3.**
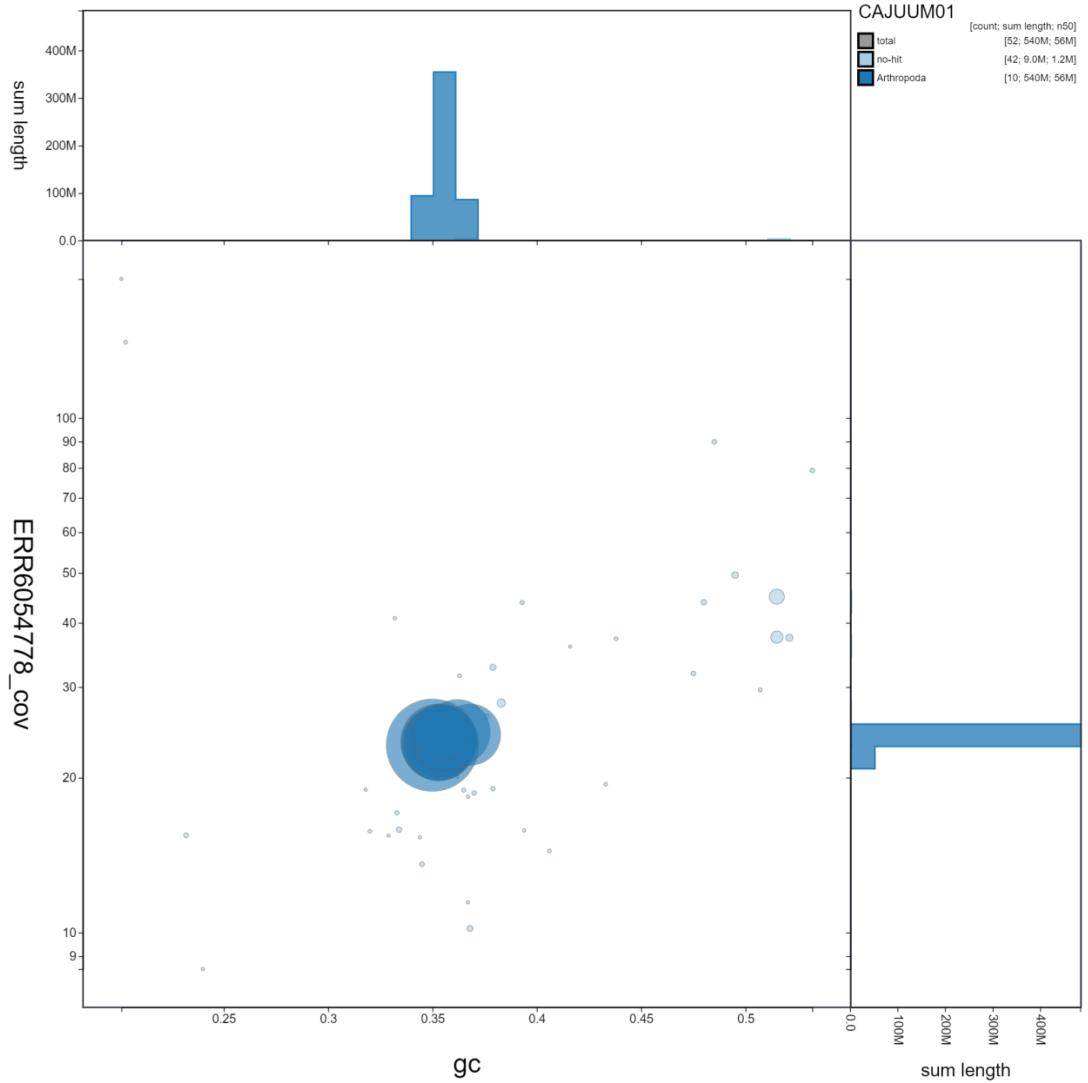
Genome assembly of *Malachius bipustulatus*, icMalBipu1.1: GC coverage. BlobToolKit GC-coverage plot. Scaffolds are coloured by phylum. Circles are sized in proportion to scaffold length. Histograms show the distribution of scaffold length sum along each axis. An interactive version of this figure is available at https://blobtoolkit.genomehubs.org/view/icMalBipu1.1/dataset/CAJUUM01/blob.

**Figure 4.**
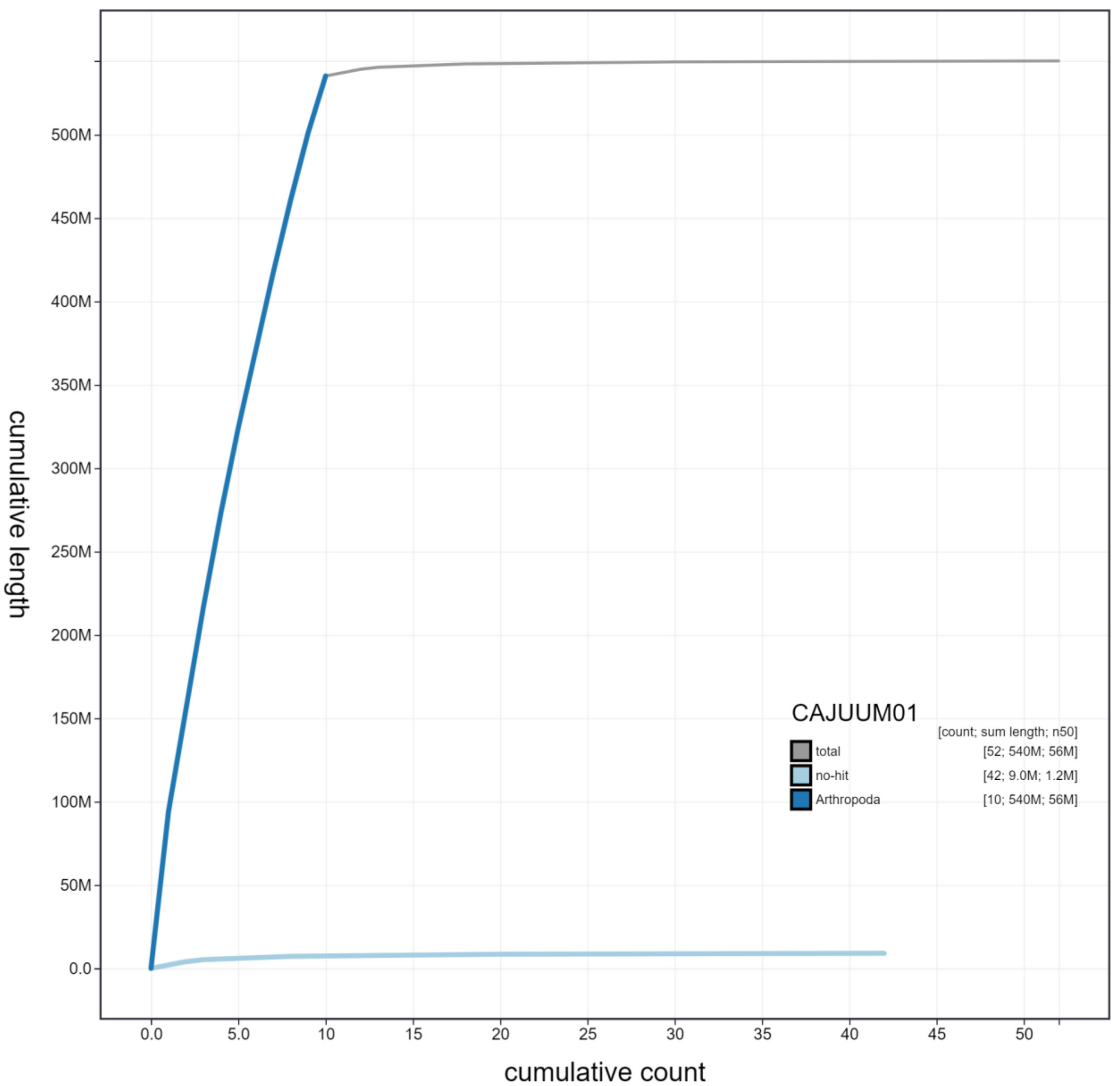
Genome assembly of *Malachius bipustulatus*, icMalBipu1.1: cumulative sequence. BlobToolKit cumulative sequence plot. The grey line shows cumulative length for all scaffolds. Coloured lines show cumulative lengths of scaffolds assigned to each phylum using the buscogenes taxrule. An interactive version of this figure is available at https://blobtoolkit.genomehubs.org/view/icMalBipu1.1/dataset/CAJUUM01/cumulative.

**Figure 5.**
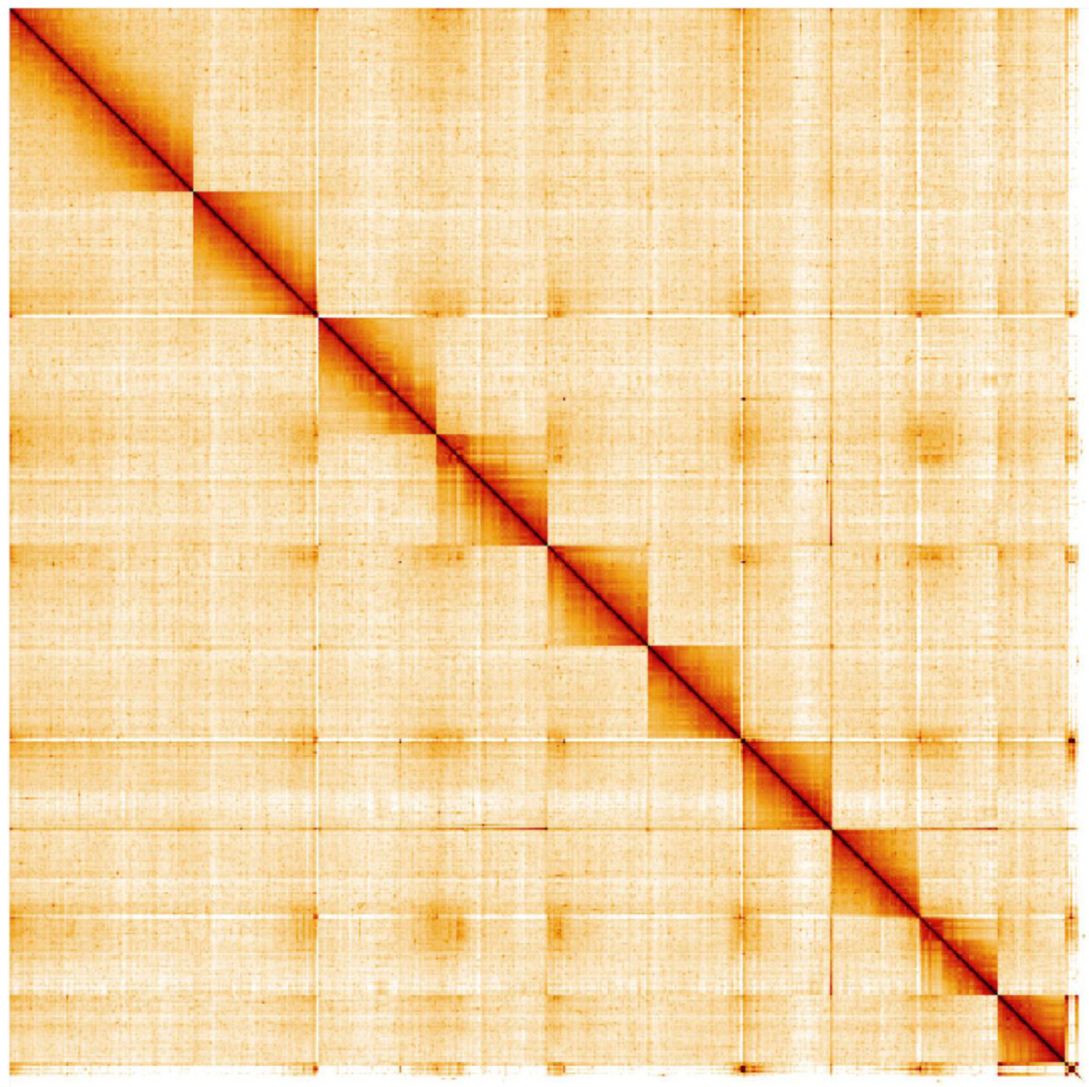
Genome assembly of *Malachius bipustulatus*, icMalBipu1.1: Hi-C contact map. Hi-C contact map of the icMalBipu1.1 assembly, visualised in HiGlass.

**Table 2.** Chromosomal pseudomolecules in the genome assembly of *Malachius bipustulatus*, icMalBipu1.1.

INSDC accession	Chromosome	Size (Mb)	GC%
OU342630.1	1	94.15	35
OU342631.1	2	62.65	35.3
OU342632.1	3	59.84	35.3
OU342633.1	4	56.30	35.4
OU342634.1	5	51.11	35.3
OU342636.1	6	45.98	36.2
OU342635.1	7	47.24	35.5
OU342637.1	8	43.55	35.6
OU342638.1	9	40.41	36.8
OU342639.1	X	34.00	35.4
OU342640.1	MT	0.02	20.2
-	Unplaced	9.01	43.1

## Methods

### Sample acquisition and DNA extraction

A single female *M. bipustulatus* (icMalBipu1) was collected from Wytham Woods, Oxfordshire (biological vice-county: Berkshire), UK (latitude 51.768, longitude -0.339) by Liam Crowley, University of Oxford, using a net. The sample was identified by the same individual, snap-frozen on dry ice and stored using a CoolRack.

DNA was extracted at the Tree of Life laboratory, Wellcome Sanger Institute. The icMalBipu1 sample was weighed and dissected on dry ice with tissue set aside for Hi-C sequencing. Tissue from the whole organism was cryogenically disrupted to a fine powder using a Covaris cryoPREP Automated Dry Pulveriser, receiving multiple impacts. Fragment size analysis of 0.01–0.5 ng of DNA was then performed using an Agilent FemtoPulse. High molecular weight (HMW) DNA was extracted using the Qiagen MagAttract HMW DNA extraction kit. Low molecular weight DNA was removed from a 200-ng aliquot of extracted DNA using 0.8X AMpure XP purification kit prior to 10X Chromium sequencing; a minimum of 50 ng DNA was submitted for 10X sequencing. HMW DNA was sheared into an average fragment size between 12–20 kb in a Megaruptor 3 system with speed setting 30. Sheared DNA was purified by solid-phase reversible immobilisation using AMPure PB beads with a 1.8X ratio of beads to sample to remove the shorter fragments and concentrate the DNA sample. The concentration of the sheared and purified DNA was assessed using a Nanodrop spectrophotometer and Qubit Fluorometer and Qubit dsDNA High Sensitivity Assay kit. Fragment size distribution was evaluated by running the sample on the FemtoPulse system.

### Sequencing

Pacific Biosciences HiFi circular consensus and 10X Genomics read cloud DNA sequencing libraries were constructed according to the manufacturers’ instructions. Sequencing was performed by the Scientific Operations core at the Wellcome Sanger Institute on Pacific Biosciences SEQUEL II and Illumina HiSeq X instruments. Hi-C data were generated using the Arima v2 Hi-C kit and sequenced on a HiSeq X instrument.

### Genome assembly

Assembly was carried out with Hifiasm ( Cheng *et al.,* 2021); haplotypic duplication was identified and removed with purge_dups ( Guan *et al.,* 2020). One round of polishing was performed by aligning 10X Genomics read data to the assembly with longranger align, calling variants with freebayes ( Garrison & Marth, 2012). The assembly was then scaffolded with Hi-C data ( Rao *et al.,* 2014) using SALSA2 ( Ghurye *et al.,* 2019). The assembly was checked for contamination and corrected using the gEVAL system ( Chow *et al.,* 2016) as described previously ( Howe *et al.,* 2021). Manual curation ( Howe *et al.,* 2021) was performed using gEVAL, HiGlass ( Kerpedjiev *et al.*, 2018) and Pretext. The mitochondrial genome was assembled using MitoHiFi ( Uliano-Silva *et al.,* 2021) and annotated with MitoFinder ( Allio *et al.,* 2020). The genome was analysed and BUSCO scores generated within the BlobToolKit environment ( Challis *et al.,* 2020). Table 3 contains a list of all software tool versions used, where appropriate.

**Table 3.** Software tools used.

Software tool	Version	Source
Hifiasm	0.12	Cheng *et al.,* 2021
purge_dups	1.2.3	Guan *et al.,* 2020
SALSA2	2.2	Ghurye *et al.,* 2019
longranger align	2.2.2	https:// support.10xgenomics. com/genome-exome/ software/pipelines/latest/ advanced/other-pipelines
freebayes	1.3.1-17-gaa2ace8	Garrison & Marth, 2012
MitoHiFi	1.0	Uliano-Silva *et al.,* 2021
gEVAL	N/A	Chow *et al.,* 2016
HiGlass	1.11.6	Kerpedjiev *et al.,* 2018
PretextView	0.2.x	https://github.com/wtsi- hpag/PretextView
BlobToolKit	2.6.2	Challis *et al.,* 2020

### Ethics/compliance issues

The materials that have contributed to this genome note have been supplied by a Darwin Tree of Life Partner. The submission of materials by a Darwin Tree of Life Partner is subject to the Darwin Tree of Life Project Sampling Code of Practice. By agreeing with and signing up to the Sampling Code of Practice, the Darwin Tree of Life Partner agrees they will meet the legal and ethical requirements and standards set out within this document in respect of all samples acquired for, and supplied to, the Darwin Tree of Life Project. Each transfer of samples is further undertaken according to a Research Collaboration Agreement or Material Transfer Agreement entered into by the Darwin Tree of Life Partner, Genome Research Limited (operating as the Wellcome Sanger Institute), and in some circumstances other Darwin Tree of Life collaborators.

## Data availability

European Nucleotide Archive: Malachius bipustulatus (common malachite beetle). Accession number PRJEB45121; https://identifiers.org/ena.embl/PRJEB45121.

The genome sequence is released openly for reuse. The *M. bipustulatus* genome sequencing initiative is part of the Darwin Tree of Life (DToL) project. All raw sequence data and the assembly have been deposited in INSDC databases. The genome will be annotated and presented through the Ensembl pipeline at the European Bioinformatics Institute. Raw data and assembly accession identifiers are reported in Table 1.

## Author information

Members of the University of Oxford and Wytham Woods Genome Acquisition Lab are listed here: https://doi.org/10.5281/zenodo.4789929.

Members of the Darwin Tree of Life Barcoding collective are listed here: https://doi.org/10.5281/zenodo.4893704.

Members of the Wellcome Sanger Institute Tree of Life programme collective are listed here: https://doi.org/10.5281/zenodo.5377053.

Members of Wellcome Sanger Institute Scientific Operations: DNA Pipelines collective are listed here: https://doi.org/10.5281/zenodo.4790456.

Members of the Tree of Life Core Informatics collective are listed here: https://doi.org/10.5281/zenodo.5013542.

Members of the Darwin Tree of Life Consortium are listed here: https://doi.org/10.5281/zenodo.4783559.
