# Supplementary material for: The genome sequence of the common malachite beetle, Malachius bipustulatus (Linnaeus, 1758)
Source: Wellcome Open Res. 2021 Nov 25;6:322. [Version 1] doi: 10.12688/wellcomeopenres.17381.1 (PMC9992895; doi:10.12688/wellcomeopenres.17381.1)
Supplement: Peer review history

# Peer review: Reviewer response for version 1

**DOI:** 10.21956/wellcomeopenres.19218.r54314

**Published:** 2023-03-07

**Authors:** Brenda Oppert

**Affiliations:** 1 USDA ARS Center for Grain and Animal Health Research, Manhattan, USA

1.  Genome sequence size - I usually refer to this as "genome assembly size" because the actual genome sequence could be different.  We measure the genome sequence size via flow cytometry and feel that is a more accurate estimation. I did check the Animal Genome Size Database and did not find an entry.  So, the genome size remains to be determined.
2. We sequence males so that we get the Y chromosome sequence (in cases where the insect has a Y chromosome) - I would expect that this insect has a Y chromosome, so I don't know why a female was used for sequencing (I don't find an explanation).
3. The assembly method is one that we also use - long read draft assembly, scaffolded by long range data.  It's assumed that all data came from the single female and thus it ensures an improved assembly.
4. The metrics of the assembly indicate that this is an adequate reference assembly.

Are sufficient details of methods and materials provided to allow replication by others?

Yes

Is the rationale for creating the dataset(s) clearly described?

Partly

Are the datasets clearly presented in a useable and accessible format?

Yes

Are the protocols appropriate and is the work technically sound?

Yes

Reviewer Expertise:

Arthropod functional genomics

I confirm that I have read this submission and believe that I have an appropriate level of expertise to confirm that it is of an acceptable scientific standard.

# Peer review: Reviewer response for version 1

**DOI:** 10.21956/wellcomeopenres.19218.r54315

**Published:** 2023-01-25

**Authors:** Duane D McKenna

**Affiliations:** 1 Department of Biological Sciences, University of Memphis, Memphis, TN, USA

This manuscript entitled ‘The genome sequence of the common malachite beetle, Malachius bipustulatus (Linnaeus, 1758)’ reports a new chromosomal-scale genome assembly of an interesting beetle in the family Melyridae (superfamily Cleroidea). The latest methods for genome sequencing, assembly, annotation, and characterization were used. The methods and sequencing results and assembly are clearly reported and interpreted, and appropriately illustrated. Nonetheless, we suggest a few minor edits (detailed below) before publication.

 BUSCO assessment of the scaffolded genome is mentioned but it is not clear whether the genome was assessed for completeness at the initial assembly step and after the removal of haplotigs. Did the BUSCO assessment show any improvements after the scaffolding step (e.g., reduced fragmented ortholog percentage)?

 It sounds the haplotypic duplications were removed after genome scaffolding. However, in reading the methods, the haplotypic duplication removal step comes before the scaffolding step.

 What led to haplotypic duplication removal? For instance, was this due to the high BUSCO duplications percentage or due to a genome profiling report suggesting that there were many duplicates in the genome?

 Is it possible to confirm that this species has 10 haploid chromosomes? All other reported chromosome counts from Melyridae range from 6 to 8 and there are species with XYp and XO sex chromosome systems (Karyotype.org).

 The date of collection of the sequenced specimen is not indicated. Also, the initial size assessment should come after the DNA extraction step.

 What parameters were used with the software tools used?

 What tools were used for contamination screening (e.g., Blast)?

 When assembling the mitochondrial genome, what sequence was used as a reference?

 Table 3 does not contain direct reference to the BUSCO software; however, they mention that they have used it.

 How was the X chromosome scaffold identified?

 Using the provided link, we can access the raw fastq files but the links to the genome assemblies are not functional or at least not apparent.

Are sufficient details of methods and materials provided to allow replication by others?

Partly

Is the rationale for creating the dataset(s) clearly described?

Partly

Are the datasets clearly presented in a useable and accessible format?

Partly

Are the protocols appropriate and is the work technically sound?

Yes

Reviewer Expertise:

Insect (especially beetle) systematics, genomics, evolution and diversity.

I confirm that I have read this submission and believe that I have an appropriate level of expertise to confirm that it is of an acceptable scientific standard, however I have significant reservations, as outlined above.

# Peer review: Reviewer response for version 1

**DOI:** 10.21956/wellcomeopenres.19218.r51932

**Published:** 2022-08-25

**Authors:** Lynette Strickland

**Affiliations:** 1 University of Memphis, Memphis, TN, USA

The report entitled, ‘The genome sequence of the common malachite beetle, *Malachius bipustulatus* (Linnaeus, 1758)’, describes the assembly of the malachite beetle using PacBio Sequel, 10X Illumina, and Hi-C data. The article is generally well-written, the methods are well-designed, and the conclusions are sound.

 Please add a short description to the Background section of why the genome assembly for this species will be useful and what future work can be addressed specifically in this system with an assembled genome resource.

Are sufficient details of methods and materials provided to allow replication by others?

Yes

Is the rationale for creating the dataset(s) clearly described?

Partly

Are the datasets clearly presented in a useable and accessible format?

Yes

Are the protocols appropriate and is the work technically sound?

Yes

Reviewer Expertise:

ecological genetics and genomics

I confirm that I have read this submission and believe that I have an appropriate level of expertise to confirm that it is of an acceptable scientific standard.
